# Reliability of digitally instructed self-reported 30-second chair stand test for lower extremity function

**DOI:** 10.1016/j.ocarto.2025.100613

**Published:** 2025-04-09

**Authors:** Leif E. Dahlberg, Oscar Karlsson, Paulina Sirard, L Stefan Lohmander, Ali Kiadaliri

**Affiliations:** a Department of Clinical Sciences Lund, Orthopaedics, Lund University, Lund, Sweden; b Arthro Therapeutics, Malmö, Sweden; c Department of Medicine and Optometry, Linnaeus University, Kalmar, Sweden

**Keywords:** eHealth, Functional test, Physiotherapy, Osteoarthritis, 30s CST

## Abstract

**Objective:**

The 30-s Chair Stand Test (30s CST), a valid and reliable test evaluating lower extremity physical function, has been integrated into a digital eHealth program. We aimed to evaluate the agreement (inter-rater reliability) between digital self-assessment and in-person physiotherapist assessment as well as intra-rater test-retest reliability of digital self-assessment among persons with hip or knee osteoarthritis.

**Design:**

Eligible participants with hip or knee osteoarthritis were identified from the digital treatment database. The 30s CST was performed through a digital self-assessment and in-person physiotherapist assessment. The inter-rater reliability study was conducted at a physiotherapy clinic and for the intra-rater test-retest reliability, the participants performed the digitally self-assessment test twice in their home.

**Results:**

The inter-rater reliability one-day study, included 18 participants (mean age 67 years and 89 ​% females) and one physiotherapist. The intra-rater test-retest, separated by 10–14 days, included 54 participants (mean age 69 years, 78 ​% females). There were, on average, 1.5 (95 ​% CI 0.6 to 2.4) more self-reported sit-to-stand repetitions for the digital self-assessment compared with in-person physiotherapist assessment. The digital self-assessment of 30s CST showed low to excellent inter-rater reliability with an intraclass correlation coefficient (ICC) of 0.87 (95 ​% CI 0.47 to 0.96) and good to excellent intra-rater test-retest reliability, ICC 0.88 (95 ​% CI 0.79 to 0.93). Bland-Altman plots suggested good levels of inter- and intra-rater reliability.

**Conclusion:**

Results suggest that the 30s CST can be measured digitally as a self-administered and self-reported measurement of lower extremity physical function in older adults with hip and/or knee osteoarthritis.

## 1 Introduction

Healthcare digitalization has been recognized as a crucial tool to handle the steadily increasing proportion of people with chronic diseases, e.g. osteoarthritis, the most common joint disease. Providing exercise therapy through digital platforms has shown similar or better outcomes compared to traditional face-to-face physical therapy but at a lower cost [[1], [2], [3]]. In transitioning from conventional physical care to digital alternatives, relevant treatments and outcome measures need to be transferred into a digital context [4].

Physical function is a key component of osteoarthritis care which can be measured using patient-reported outcome measures or performance-based tests [5]. Current evidence suggests that these two approaches may capture different aspects of functional abilities and suggests the use of both for a comprehensive assessment of physical function in osteoarthritis [6]. However, perceived difficulty in measuring physical function, especially performance-based tests, remotely via digital tools imposes challenges in the widespread adoption of these tools in hip and knee osteoarthritis care [7]. Indeed, there is limited evidence on validity and reliability of digital self-assessment of performance-based tests among persons with osteoarthritis. Video-based assessments of physical performance tests were shown to be valid and reliable for measuring physical function among adults with knee osteoarthritis via Microsoft Teams® [8]. However, of particular interest in the context of the growing role of eHealth in osteoarthritis management, is to examine the reliability of digital self-assessment of physical function in self-supportive digital treatment programs. We aimed to assess reliability of the 30-second Chair Stand Test (30s CST) as it is used in a digital education and exercise program (the Joint Academy®) for patients with knee and hip osteoarthritis [9]. The 30s CST is a widely used physical function test to evaluate lower extremity function recommended by the Osteoarthritis Research Society International [10]. Specifically, the purpose of the study was to find out if the test as used in the program, under real life, realistic conditions is suitable for the purpose of measuring individual changes over time by evaluating: 1. The agreement between the participant's digital self-assessment and the in-person physiotherapist's assessment of the 30s CST and 2. Participant's intra-rater test-retest reliability of digital self-assessment of the 30s CST. We hypothesized that digital self-assessment of 30s CST would be reliable to assess physical function in people with knee or hip osteoarthritis.

## 2 Method

Ethics approval was obtained through the Swedish Ethical Review Authority (Dnr: 2022-06040-02) and informed consent was provided by all participants. Participants’ inclusion criteria were: age 55 years or older to reflect the typical population treated in the digital program, a clinical diagnosis of hip and/or knee osteoarthritis based on criteria outlined by NICE [11], having participated for at least 6 weeks in the digital treatment to get used to the app, and ending the digital treatment more than 6 months prior to the present study. The digital program delivered as an app, based on peer-reviewed published evidence, delivers daily video exercises, text lectures, and self-management support. Participants are supervised by a dedicated physiotherapist via telephone, video calls and by asynchronous chat communications in the digital program [9].

Eligible participants for the present study were identified from the digital treatment database. Inclusion was on a first-come basis. Two separate studies were conducted: 1. The agreement between the participant's digital self-assessment and the in-person physiotherapist's assessment of the 30s CST and 2. Participant's intra-rater test-retest reliability of digital self-assessment of the 30s CST. Participants could only take part in one of the two studies.

The sample size for the inter- and intra-rater reliability studies were determined based on previous studies [12,13]. For example, for an acceptable ICC of 0 and an expected ICC of 0.7, an alpha value set at 0.05 and a minimum power of 90 ​%, a sample size of 13 is required to test for reliability studies [12].

The 30s CST was performed on a 45 ​cm high chair with the back against a wall. Initially, the participants were guided and instructed by a physiotherapist how to perform the 30s CST [14]. They then practiced the test once. Next, to perform *digital* self-assessment 30s CST as it is used in the digital program, the participants downloaded the app to a smartphone to receive text and video instructions on how to perform the test, identical to those in the digital program [15]. The instructional video shows that the mobile phone should be placed leaning against a couple books on a table before activating the timer in the app. The timer counted down from 30 ​s, while the participant simultaneously performed and counted the number of sit-to-stand repeats. There was no alarm at the end of the countdown. The physiotherapist monitored and reported the number of in-person repetitions.

The inter-rater reliability study was conducted on the same day at a physiotherapy clinic. The participants were randomized into two groups. One group performed the digital 30s CST and self-reported the number of repetitions, and the other group performed the traditionally 30s CST first with one single physiotherapist calculating and reporting the number of sit-to-stand repeats. The groups then switched in a crossover design to minimize the risk of learning bias. Between the tests the persons had a 20 ​min rest period during which they answered the questionnaires [16].

To assess intra-rater test-retest reliability, the participants performed the digital self-assessment test twice in their home environment instructed by the downloaded app via text and video instructions as shown by screenshots (Supplement). There was a 10–14 day interval between the two test sessions. The interval was chosen to be similar to the treatment program, where there is a 10–14 days interval between each 30s CST and to be in accordance with a 1–2 weeks interval used in a previous study examining reliability of functional tests delivered face-to-face and via telehealth [17]. Moreover, a previous study exploring the intra-rater test-retest reliability of several functional performance tests including 30s CST among individuals with hip OA found 2-week interval to yield the highest reliability for 30s CST [18].

Self-reported data was collected prior to performing the testing including age, sex, body mass index (BMI, kg/m2), pain using a numerical rating scale (NRS 0–10 where 0 ​= ​no pain and 10 ​= ​worst pain imaginable), disability using the Knee/Hip disability and Osteoarthritis Outcome Score 12 (KOOS-12/HOOS-12, scale 0–100 where 0 ​= ​extreme joint problems, 100 ​= ​no joint problems) [19] and activity impairment via a subscale of the Work Productivity and Activity Impairment questionnaire asking “During the past seven days, how much did your health problems affect your ability to perform your normal daily activities, excluding your job?” with a numerical rating scale (0–10, best to worst) [20].

### 2.1 Analysis

The intraclass correlation coefficients (ICC) with 95% confidence intervals (CI) were used to assess inter- and intra-rater reliability. We estimated the ICC for inter-rater reliability using two-way random effects model and the ICC for intra-rater reliability using two-way mixed effects model [21]. For both ICCs, we employed the setting with single rater and absolute agreement. The ICC was interpreted as low (<0.5), moderate (0.5–0.74), good (0.75–0.9) or excellent (>0.9) [21]. Additionally, we measured the measurement error using the standard error of measurement (SEM) calculated as standard deviation of measurements multiply the square root of (1-ICC) [22]. Minimal detectable change (MDC_ind_) is the smallest within-person change that can be detected by the test beyond measurement error and was calculated as SEM∗1.96∗square root of 2 [22]. The group level MDC (MDC_group_) can be calculated by dividing the individual level MDC by the square root of the sample size [22]. We constructed Bland-Altman plots with 95% Limits of Agreement to assess the agreements [23]. The mean (95% CI) difference between two digital self-assessments as well as between digital self-assessment and in-person physiotherapist assessment were computed using paired t-tests. To compare the baseline characteristics of those with and without missing responses, we computed standardized mean difference (difference in mean divided by the square root of the average variance) and considered an absolute difference ≥0.1 as meaningful difference [24]. Data was analysed using Stata v.18.

## 3 Results

### 3.1 Inter-rater reliability

A total of 18 participants, 89% females, with a mean (SD) age 67 (6) years were included (Table 1). The mean NRS pain and activity impairment suggested mild OA severity.

**Table 1:** Patient characteristics of samples included in inter- and intra-rater reliability studies.

	Inter-rater reliability	Intra-rater test-retest reliability		
		Included	Excluded	Absolute SMD
N	18	44	10	
Female sex, n (%)	16 (88.9)	34 (77.3)	5 (50.0)	0.57
Age, mean (SD)	67.1 (5.9)	68.6 (6.7)	70.9 (7.2)	0.33
Osteoarthritis site, n (%)				0.06
Knee	14 (77.8)	32 (72.7)	7 (70.0)	
Hip	4 (22.2)	12 (27.3)	3 (30.0)	
Body mass index, mean (SD)	26.6 (4.5)	26.5 (4.0)	25.8 (4.2)	0.15
NRS pain (0–10, best to worst), mean (SD)	3.5 (2.2)	4.1 (2.5)	3.2 (2.3)	0.37
Activity impairment (0–10, best to worst), mean (SD)	2.6 (2.7)	3.0 (2.4)	2.4 (2.1)	0.25
KOOS-12/HOOS-12 (0–100, worst to best), mean (SD)	64.1 (15.8)	64.5 (19.0)	73.1 (15.7)	0.50

SMD: Standardized mean difference, SD: Standard deviation, NRS: Numerical rating scale.

**Fig. 1:**
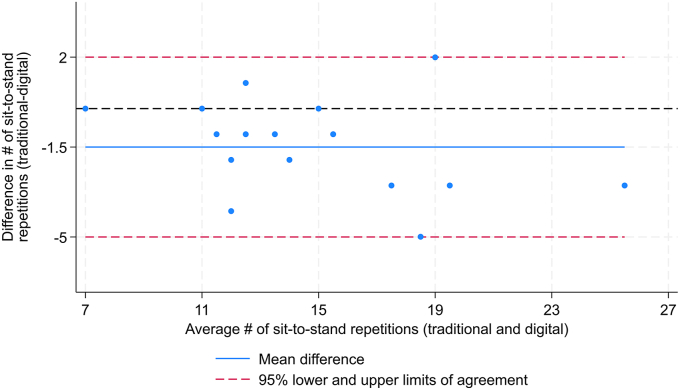
Bland-Altman plot of the agreement between digital self-assessment and in-person physiotherapist assessment 30s chair stand test. The blue line denotes the mean difference, and the dashed red lines represent the LoA range of 2 to −5. The dashed black line represents the line of perfect average agreement.

The inter-rater reliability between the digital self-assessment and in-person physiotherapist assessment of 30s CST was low to excellent with ICC of 0.87 (95% CI 0.47 to 0.96). The estimated SEM, MDC_ind_ and MDC_group_ were 1.6, 4.4, and 1.0 repetitions, respectively. When participants self-assessed their test digitally, they performed, on average, 15.8 (SD 4.7) sit-to stand repetitions, whereas when observed by a physiotherapist they did 14.3 (SD 4.0) sit-to-stand repetitions. As a result, there were, on average, 1.5 (95% CI 0.6 to 2.4) more reported sit-to-stand repetitions when the test was self-assessed. The Bland-Altman plot showed that all individual 30s CST values were within the LoA, with no evidence of systematic bias (Fig. 1).

### 3.2 Intra-rater test-retest reliability

A total of 54 participants, 78% females, with mean (SD) age 69 (6) years were included. Of these, ten did not complete re-test despite reminders and were therefore excluded. Those included were younger, with a higher proportion of females, a higher body mass index, and greater OA severity compared with those excluded (Table 1).

**Fig. 2:**
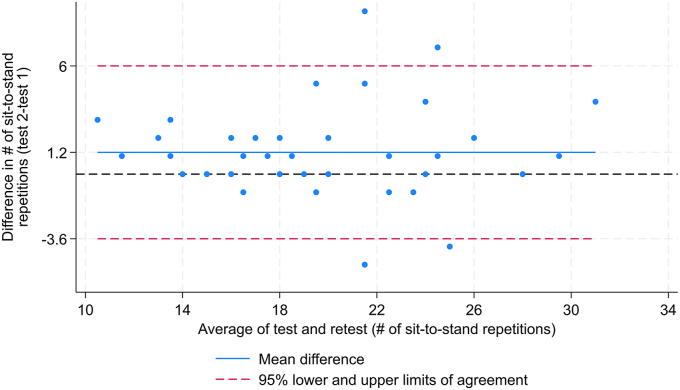
Bland-Altman plot of the agreement between the first and second digital self-assessment 30s chair stand test. The blue line denotes the mean difference, and the dashed red lines represent the 95 ​% LoA. The dashed black line represents the line of perfect average agreement.

There was a good to excellent agreement between the two digitally self-reported tests with an ICC of 0.88 (95% CI 0.79 to 0.93). The estimated SEM, MDC_ind_ and MDC_group_ were 1.7, 4.8 and 0.7 repetitions, respectively. Compared with the first test, the participants at the second test reported on average 1.2 (95% CI 0.5 to 1.9) more sit-to-stand repetitions. The Bland-Altman plot showed a good level of agreement between test and retest values with only four (9%) observations outside the 95% LoA, and no evidence of systematic bias (Fig. 2).

## 4 Discussion

The digital self-assessment of the 30s CST showed, on average, good inter- and intra-rater reliability. The findings suggest that the 30s CST can be administered digitally as a self-test to reliably measure physical function in people with knee or hip osteoarthritis.

The inter- and intra-reliability of in-person assessment of 30s CST by external observer in persons with osteoarthritis is well established with ICC>0.9 [18,25,26]. Several studies also established the inter- and intra-rater reliability of digitally administered 30s CST assessed by external observer or accelerometer in healthy adults [27], persons with osteoarthritis [28] and other diseases [[29], [30], [31]]. However, less is known about the reliability of 30s CST as a self-test in general and among people with osteoarthritis. Specifically, to our knowledge, only a recent study investigated the inter- and intra-rater reliability of 30s CST administered *non-digitally* as a self-test among persons with osteoarthritis and reported excellent intra-rater and good inter-rater reliability [32]. Consistent with these previous studies, we observed good inter- and intra-rater reliability for digital self-assessment of 30s CST. Taking together, these results support the use of 30s CST as a self-test for assessment of physical function in older adults with hip or knee osteoarthritis in a digital treatment context.

In the present study, the participants self-reported on average 1.5 more sit-to-stand repeats compared to that of external observer, and in repeated tests participants made on average 1.2 more sit-to-stand repeats in the second test than in the first. While statistically significant, the clinical relevance of these differences is uncertain, given that the estimated minimal clinically important difference of the 30s CST is a 2.0–2.6 repetitions difference [33]. Regarding individual changes in 30s CST, a minimum detectable change for an individual was suggested to be between 2.3 and 2.8 sit-to-stands [26]. Moreover, these differences were slightly smaller than the estimated standard error of measurement in our study suggesting that these observed differences are likely due to measurement error rather than actual differences/changes. Albeit the observed differences for some of the retest 30s CST values were skewed or outside these intervals. An explanation for a deviation in any direction may be the need to look at the timer and count the number of sit-to-stand at the same time. The overestimation of the individual's number of sit-to-stand is not likely to be significant in clinical studies.

The observed difference in test-retest results may also suggest a learning effect consistent with findings from previous research investigating the traditional 30s CST [25,26]. A possible risk of learning effect suggests that the 30s CST should not be used as a basis for introducing more challenging exercises during patients' progress in the digital program. Progress to more demanding exercises is better to be based on patients’ own evaluation of perceived difficulty of the performed exercises [9]. To better control for individual differences in getting used to the 30s CST, future studies may consider including a participant practice session before starting the test-retest study.

Several limitations of the present study should be acknowledged. The design of the study, to find out if the 30s CST as used in the program was suitable to detect individual changes in function over time, limited our ability to identify reasons for why the participants self-reported more sit-to-stand repeats compared to that of external observer, and more sit-to-stand repeats in the second test than in the first test. The 95% CI for ICC for assessing inter-rater reliability was wide, which may in part reflect the small sample size in the study. Specifically, formal sample size calculations were not implemented for the present study and the sample size from previous studies were used. In comparison, a recent study assessing inter-rater reliability of 30s CST as self-test included 118 participants and despite slightly lower ICC (0.81 vs. 0.87 in the present study), it had narrower 95% CI (0.72, 0.87) [32]. In the intra-rater test, the time between the test and retest was separated by 10–14 days in line with the digital treatment program. While this time interval might seem long for a test-retest assessment, Bieler et al. [18] investigated the reliability of the traditional 30s CST (and four other functional performance tests) using different time intervals and found that two-week interval yielded the highest 10.13039/501100018804ICC value for 30s CST, supporting our study's chosen time interval. This time interval has also been applied in other studies including a recent study among persons with lower limb musculoskeletal disorders [17,34]. Despite this, possible changes in factors influencing physical function (e.g. pain intensity) over this period and their effects on test-retest reliability cannot be ruled out. The inter-rater reliability study was conducted in a controlled environment at a physiotherapy clinic with a single physiotherapist present. Results of a test performed at home may have yielded greater individual differences as compared to that of a controlled environment. For example, the height of the chair seat may not be adjustable to fit the individual participant and adherence to details of test instructions is unknown [35,36]. The presence of only one physiotherapist observer in the study prevented us from assessing the reliability of physiotherapist assessments. At the time of the study, the physiotherapist involved in the study had approximately three years of experience administering the traditional 30s CST and around four years of experience overseeing the digitally administered 30s CST. The participants had participated in a first-line OA treatment including exercises more than 6 months prior to the present study. Accordingly, they were selected from a previously treated OA population which limits the generalizability of our findings to the general OA population, specifically with respect to participants unfamiliar with digital tools. Future studies need to assess the reliability of digital self-assessment in a more diverse population. The differences between included and excluded participants in the test-retest may introduce bias. However, considering the small to moderate magnitude of the standardized mean differences between these groups [37], it is unlikely to change the overall interpretation of results. Notably, the excluded individuals reported better patient-reported outcomes and slightly higher number of 30s CST at the first test measurement compared with those included (19.6 repetitions vs. 18.7 repetitions).

In conclusion, our results suggest that the 30s CST can be digitized for reliable self-assessment of lower extremity physical function in older adults with hip and/or knee osteoarthritis. Furthermore, the 30s CST may be a reliable self-administered and self-reported measure of physical function as used in the app program.

## Author contributions

OK contributed to the conception and design of the study, collection and assembly of data, drafting the article, and revising it critically. AK contributed with statistical expertise and interpretation of the data, drafting, and critical revision of the article. LED and PS contributed to the conception and design of the study, interpretation of the data, drafting of the article, critical revision of the article and obtained funding for the study. LSL contributed with interpretation of data, critical revision of the article for important intellectual content. All authors provided final approval of the version to be submitted. LED (leif.dahlberg@med.lu.se) take responsibility for the integrity of the work as a whole.

## Role of funding source

Support for this study was provided by the 10.13039/501100006075Greta and Johan Kock Foundation and Stiftelsen för bistånd åt rörelsehindrade i Skåne. The study sponsor had no role in the study design, data collection, analysis, interpretation, manuscript writing, or the decision to submit the manuscript for publication.

## Declaration of competing interest

AK acts as part-time scientific advisor to Joint Academy®. LSL acts as part-time scientific advisor to Joint Academy®, and as a DSMB member for The Arthritis Foundation Inc. and Arthrex. PS is employed by Joint Academy®. OK is a physical therapist at Joint Academy®: LED is co-founder and chief medical officer at Joint Academy®.
