# Supplementary material for: Reliability of digitally instructed self-reported 30-second chair stand test for lower extremity function
Source: Osteoarthr Cartil Open. 2025 Apr 9;7(2):100613. doi: 10.1016/j.ocarto.2025.100613 (PMC12053707; doi:10.1016/j.ocarto.2025.100613)
Supplement: Multimedia component 1 [file mmc1.pdf]

Instructions for conducing the digital self-assessment test at home.

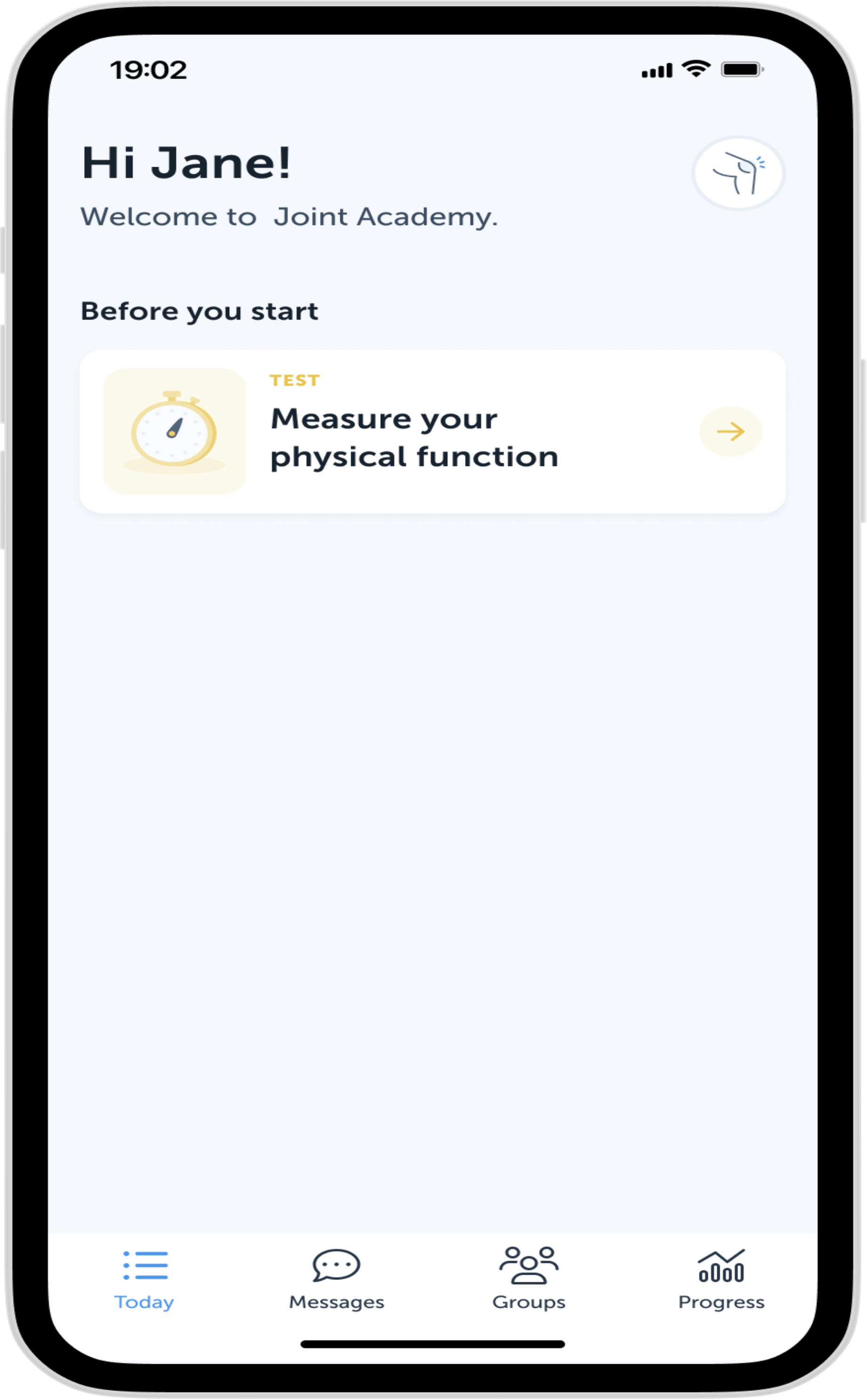

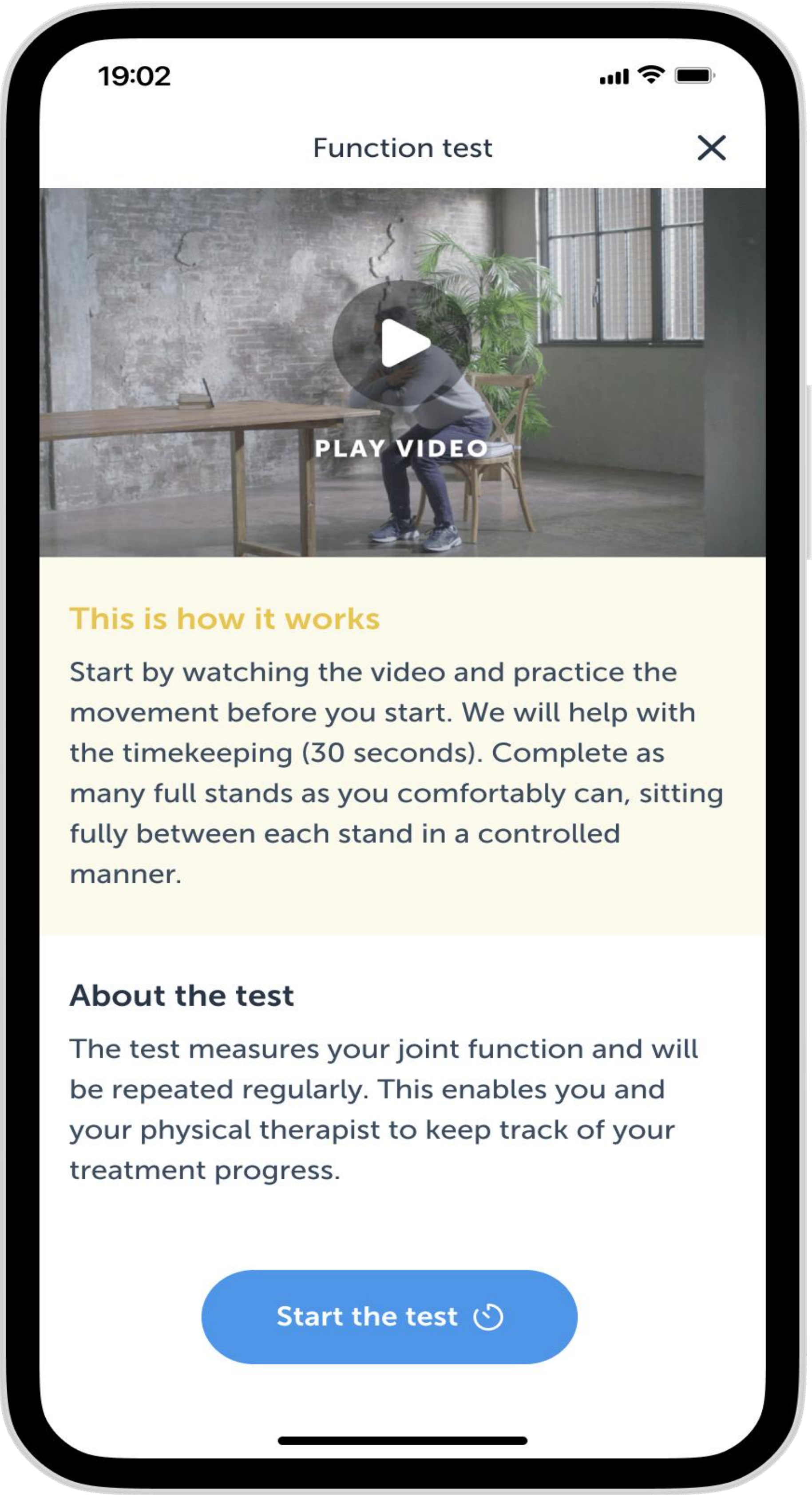
19:02
Function test
PLAY VIDEO
This is how it works
Start by watching the video and practice the movement before you start. We will help with the timekeeping (30 seconds). Complete as many full stands as you comfortably can, sitting fully between each stand in a controlled manner.
About the test
The test measures your joint function and will be repeated regularly. This enables you and your physical therapist to keep track of your treatment progress.
Start the test

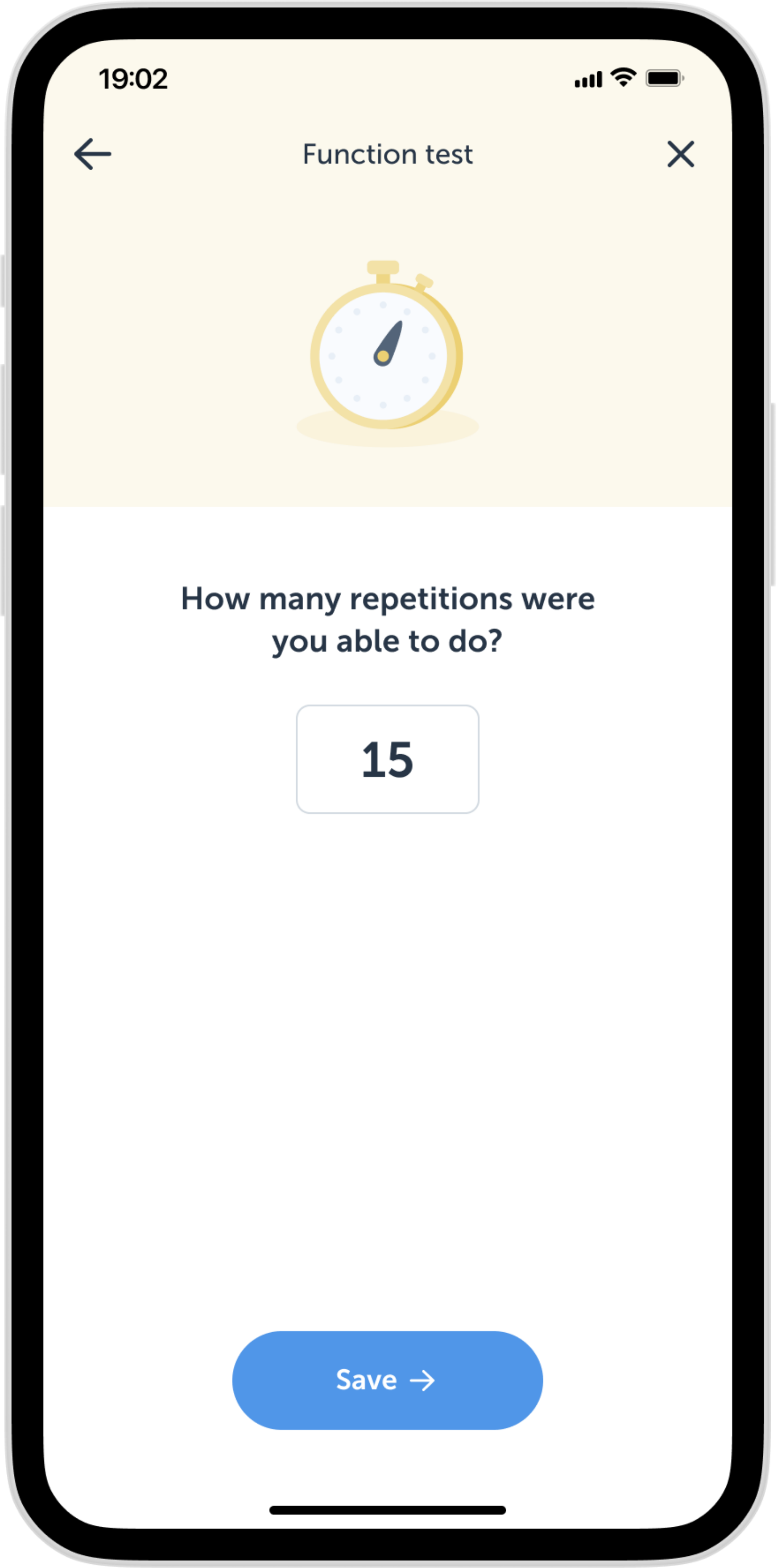
19:02
Function test
How many repetitions were
you able to do?
15
Save
